# Selfish personalities influencing start-up intention and motivation: a study of Vietnam

**DOI:** 10.1186/s13731-022-00208-5

**Published:** 2022-02-15

**Authors:** Tran Thi Hong Lien, Tran Tu Anh, Truong Nhat Anh, Le Huu Tuan Anh, Ngo Thi Thien Thao

**Affiliations:** 1 Faculty of Business Administration, University of Economics and Law, Vietnam National University - Ho Chi Minh City, 669 National Road No.1, Quarter 3, Linh Xuan Ward, Thu Duc, Ho Chi Minh City, Vietnam; 2 Faculty of Accounting and Auditing, University of Economics and Law, Vietnam National University, Ho Chi Minh City, Vietnam

**Keywords:** Selfish personality, Dark triad, Entrepreneurship, Start-up, Vietnam

## Abstract

This research explores the influences of selfish personalities of the Dark Triad on start-up intention and motives based on a sample of 400 university students in Vietnam, discovering mixed effects of narcissism, psychopathy, and Machiavellianism. A high level of narcissism and Machiavellianism leads to high start-up intention. There is a negative relationship of Machiavellianism with pro-social motive and a positive association with selfish entrepreneurship. In addition, narcissism is positively associated with pro-social start-up motives. This study has found no effect of psychopathy but a positive link to selfish entrepreneurial motivation. Implications have been suggested for educators and investors.

## Introduction

Entrepreneurship is substantially appreciated as an indispensable component of socio-economic development, because it significantly contributes to economic survival and welfare, employment, and scientific advancement (McMullan et al., 1986; Obschonka et al., 2017). Countries take advantage of entrepreneurship to increase employment and reduce the poverty rate sharply (Neneh, 2019), especially under the tremendous impact of technology development on venture creation (Briel et al., 2017). Moreover, in the blooming context of the world start-up economy (Startup Genome, 2020), young generations, including undergraduates, are motivated and orientated to establish business early in their life.

The number of Vietnam start-up companies has skyrocketed for the last 10 years, and the country has earned the top 5 ecosystem position in the Southeast Asian region (Cento Ventures, 2021). This achievement is the result of the country’s economic development process and government support policies, such as “Support Innovative Start-up Ecosystem in Vietnam until 2025” National Program (ISEV) (Vietnam Ministry of Science & Technology, 2018). According to the ESP Investment Fund and Cento Ventures report, Vietnam ranks third in its people’s positive attitude towards entrepreneurship and the growth of creative entrepreneurial ecosystems in the Asian region (VnEconomy, 2021). However, several scams have happened in real estate, forex trading, and multi-level sales; thousands of victims have lost all their assets to the scammers. The context raises a question of which motive has driven people to commit to such inhumane start-ups and whether internal causes such as personalities play a role.

The linkage between psychological personality and entrepreneurship has been widely investigated (Antoncic et al., 2015; Hamilton et al., 2019; Şahin et al., 2019). These articles generally applied the Big Five model when examining a person’s characteristics, which are more likely to be on human’s ‘good’ side. Cooper and Artz (1995) argued that self-employment is driven by moral motives—inspiration for participating in activities that produce positive results or resolve others’ problems. On the other side, Hmieleski and Lerner (2016) stated that the dark side of people affects their intention to start a business and counterproductive motives. The dark side refers to three negative personalities of Machiavellianism, narcissism, and psychopathy. The results are not consistent between the two groups of undergraduates and postgraduates. Recently, some research in the Western context additionally examined the ‘hidden’ side of an entrepreneur (DeNisi, 2015; Kraus et al., 2020; Tucker et al., 2016). However, limited research investigates this relationship in Asian cultures. The gap is a lack of literature on selfish or negative personalities, entrepreneurial intention, and motivation in the Vietnam context.

This paper aims to fill the gap using data from an online survey with undergraduates in Vietnam universities and structured equation modeling (SEM) analysis. The contribution of this research is to evaluate the influences of the three selfish personal traits on the students’ entrepreneur’s intentions and motives to start a business in a new context of an emerging economy. On that basis, the authors offer some implications for relevant stakeholders to help improve students’ entrepreneurial capacity in Vietnam. The following section is a literature review followed by methodology, results, discussion, implications, limitations, and conclusion.

## Literature review and hypothesis development

### Entrepreneurial intention, motives and selfish personalities

The intention is an effective predictor of behavior (Ajzen, 2011), and entrepreneurial intention guides individuals' efforts to establish their own business (Do & Dadvari, 2017; Thompson, 2009). Individual variables (i.e., age, gender, education, family background, and education) influence people’ start-up intention (Fatoki, 2014; Hatak et al., 2015; Quan, 2012; Smith et al., 2016); however, personality is considered to have a more significant impact (Crant, 1996). Personality is a distinctive attribute of considerations, sentiments, and practices that are used to distinguish humans. It internally forms, develops, and remains over a lifetime. As there are various ways to form a unique personality, many researchers came up with professional systems and models to measure typical personalities, such as the Big Five Model, Myers Briggs Type Indicators and Dark Triad. Regarding entrepreneurial intention, negative personalities may be influential, in addition to popular attributes of an entrepreneur, such as the determination to act, innovativeness, and risk-taking (Littunen, 2000).

A selfish individual is someone who concerns excessively or exclusively for himself or herself and is likely to take selfish behaviors. As a popular term in psychology, the Dark Triad with three elements of narcissism, psychopathy and Machiavellianism is a typical example of selfish personalities (Paulhus & Williams, 2002), leading to selfish behaviors, such as gambling other people’s money (Jones, 2013). The selfish characteristics are socially harmful (Jonanson & Tost, 2010). They can be different but share the similarities of egocentric, cruel, and destructive nature (Jonason & Webster, 2010) and are associated with selfish behaviors, such as acting dishonestly to take advantage of other people (Furnham et al., 2013).

Individuals with strong Machiavellian tendencies have a self-centered desire and a high possibility of manipulating others to achieve their personal objectives (Haynes et al., 2015). They generally show no emotion with things that happen around them, only care about their self-interest, and hardly concern about the consequences. Narcissists prefer the feelings of privilege and an inflated sense of their importance and ignorance of others (Twenge et al., 2008). To maximize their wealth (Boddy, 2006, 2015), people with psychopathy can take anti-social activities that violate norms and conventions (Boddy, 2014) to gain control power (Deutschman, 2007) over their desired short-term benefits.

Kramer et al. (2011) found a positive and significant influence of the selfish Dark Triad on start-up intention. According to Baumol (1996), entrepreneurial activities can be classified into three categories of productive, unproductive, and destructive. Regardless of an entrepreneur having productive or unproductive motives, their decisions impact on the business orientation and results as well as national economic growth (Sauka & Welter, 2007). On the one hand, productive entrepreneurial motives induce a positive value creation process for society (Baumol, 1996). These motives are more likely to engage in win–win situations for all parties. The vital activity that an entrepreneur with productive or pro-social entrepreneurial motives possesses is establishing a legitimate organization, which has a proper certificate of business registration for an organization aligned with the code of law (Sauka, 2008). Pro-social entrepreneurial motives are not a temporary action but a series of activities that create positive values for people and society, especially in a long time, facilitating the prediction of succeeding works (Acs et al., 2013).

On the other hand, unproductive or selfish relationships happen when there is an imbalance between values brought by parties (Cook et al., 2013). This relationship is a highly zero-sum game, which contrasts with the win–win situation in the pro-social entrepreneurial motives. To decrease other people’s prosperity, selfish entrepreneurs tend to take activities, such as rent-seeking and manipulation (Baumol, 1996; Sauka, 2008). Moreover, the businesses of these people have a significant association with illegal and ‘black' activities (e.g., drugs, deception, and extortion), which goes against social norms (Sauka, 2008).

### Social exchange theory and life history theory

Venturing is a profit-making process by utilizing resources of which human relations represent an essential type of capital in their social context. The social exchange theory implies that a relationship's worth is the difference between the give and the received (Cook et al., 2013). According to O’Boyle (2012), individuals are more likely to build and focus on relationships that they acquire maximum welfare with minimum or zero expenditures, which comes up with the idea of social exchange theory. These individuals have a desire for temporary relationships to take all advantages as they can and abandon these “friends” for acquiring new resources from “new friends.” Life history theory explains individuals' social behavior, which was derived from general evolutionary theory. The combination of personality traits with life history perspectives (Wilson, 1975) illustrates the chances of fast strategy (focus on immediate or short-term outcomes) or slow strategies (focus on survival, long-term investment) a personality might present. Indeed, people with Dark Triad personality traits have strong motivation to follow the fast-life strategy (Jonason et al., 2015) and start a new venture (Hmieleski & Lerner, 2016). These two theories are the foundation based on which the following hypotheses are developed; and a summary of previous studies is presented in Table 1.

**Table 1:** Overview of previous studies

Author (year)	Start-up intention and motivation (independent variable)	Selfish and other personality indicators	Theory applied	Country
Altinay et al. (2012)	Entrepreneurial intention	Family tradition (+) Locus of control (insignificant) Innovativeness (+) Propensity to take risks (+) Tolerance of ambiguity (insignificant) Need for achievement (insignificant)	McClelland’s motivation theory	United Kingdom
Antoncic et al. (2015)	Entrepreneurship (activity and propensity)	Openness (+) Conscientiousness (insignificant) Extraversion (+) Agreeableness (+) Neuroticism (insignificant)	The discovery theory of entrepreneurial action	Slovenia
Brunell et al. (2008)	Emergent leadership	Narcissism (+)	Theory of emergent leadership	United States
Chen et al. (1998)	Entrepreneurial intentions	Entrepreneurial Self-efficacy (+)	Social learning theory Expectancy theory	United States
Crant (1996)	Entrepreneurial intentions	Proactive personality scale (+)	Interactionist theory	United States
Haynes et al. (2015)	Financial success of young start-up	Greedy leader (–) Hubristic leader (−)	Theory of greed and hubris	United States
Hmieleski and Lerner (2016)	Entrepreneurial intentions	Narcissism (+) Psychopathy (insignificant) Machiavellianism (insignificant)		United States
	Unproductive entrepreneurial motives	Narcissism (insignificant) Psychopathy (+) Machiavellianism (+)	Life History Theory and Social Exchange Theory	
	Productive entrepreneurial motives	Narcissism (insignificant) Psychopathy (+) Machiavellianism (insignificant)		
Littunen (2000)	Entrepreneur's personality characteristics	Entrepreneurship (+)	McClelland’s theory Rotter’s locus of control theory	Finland
	Achievement motivation	Entrepreneurs' co-operation (−) Entrepreneurs' personal interest network (+)		
	Control of powerful others	Entrepreneurs' co-operation (−)		
Mathieu and St-Jean (2013)	Entrepreneurial Intentions	Narcissism (+)	Career choice theory Person-environment fit theory	Canada

### Hypotheses

Narcissism is the trait that received the most intense discussion (Kraus et al., 2020). It has been explored as having a positive association with the start-up intention (Cai et al., 2021; Hmieleski & Lerner, 2016). According to Mathieu and St-Jean (2013), narcissism is positively associated with self-efficacy, locus of control, and risk-taking. Moreover, narcissistic people are dominant and always desire for fame (Boddy, 2015; Jonason et al., 2012). Hence, start-up intentions have been assumed as a respected and seductive professional decision (Magister, 2013), congruent with the self-claimed importance of narcissists. Entrepreneurs are higher achievement-oriented in their actions than non-entrepreneurs (Kollmann et al., 2007). As ambition to own achievement is an aspect of narcissism, perhaps this personality is one of the motivations for a person to start their own business.

According to social exchange theory, self-interest activities widely exist within the economic field, where competition and rapacity manipulate the actions of individuals (Ekeh, 1974). Roloff (1981) commented that self-interest is not a negative thing if it is recognized and becomes the pointer of interpersonal relationships to promote both parties' self-interest. However, this positive aspect is not familiar with narcissists who strive for immediate benefits by acting in ways harmful to their counterparts. Besides, a person with a high level of narcissism exhibits anti-social behaviors. In the role of principals, narcissists only care for their power and go against criticism (Resick et al., 2009). These characteristics make narcissistic-inclined people defy many actions to achieve their own self-interested business goals and do not bring many economic benefits to society and related parties, such as staff, partners, customers. These arguments suggest three sub-hypotheses H1 (a, b, c).

#### H1

*Individuals’ levels of narcissism have a positive impact on their start-up intention (H1a), selfish start-up motives (H1b) and a negative impact on their level of pro-social start-up motives (H1c).*

Machiavellianism is considered a factor that positively influences the intention to establish a new business (Cai et al., 2021; Hmieleski & Lerner, 2016). As Machiavellians represent the “darkest” and the most selfish characteristics, with which they can exploit other people to satisfy their interests (Li et al., 2020; Wenzhi et al., 2017) with self-motivation. Besides, Zettler et al. (2011) commented that Machiavellians have a high demand for results and a strong determination to achieve goals. Owning and running a business is a visible achievement which are well-recognized by the society, and because of the attractive target of Machiavellians, it gives them the feeling of possession, power, and wealth (Hmieleski & Lerner, 2016; Rapp-Ricciardi et al., 2018).

Do and Dadvari (2017) point out that people with Dark Triad personality traits in general, and Machiavellianism in particular, evaluate the future as uncertain and unforeseeable, so they favor a fast-life strategy to satisfy their immediate needs and achieve quick gains. As focusing on short-term results can provide Machiavellians with more certain benefits, they tend to ignore the uncertain long-term resource investments (Jonanson & Webster, 2010). Therefore, the new destructive business orientation is aggressively competing for individual rewards, which replaces cooperation between parties to increase investment returns of both sides. With a tendency to exploit people to gain self-interest by conducting non-value-added work (Dahling et al., 2009), Machiavellians are less likely to be aware of business ethics (Simmons et al., 2013). From the earlier empirical evidence, it could be inferred that Machiavellians have a strong incentive to start a new business, but they are likely to engage in crafty, valueless business practices for the society as in the hypotheses H2 (a, b, c) below.

#### H2

*Individuals’ levels of Machiavellianism have a positive impact on their start-up intention (H2a), selfish start-up motives (H2b), and a negative impact on their pro-social start-up motives (H2c).*

Psychopathy is a psychological trait that its possessors often loathe social standards and possess amusement to oppose norms (Mathieu & St-Jean, 2013). Psychopaths may reach high social positions as productive leaders who are intelligent and charismatic (Brunell et al., 2008). In addition, successful managers and entrepreneurs have a higher psychopathy level than others. The overconfidence in their intellect pushes sympathy away from psychopaths (Kramer et al., 2011) and the lack of sympathy induces them to engage in unethical and violent actions on the way to their goals (Cai et al., 2021). Besides, psychopaths have a strong desire for self-promotion and impression by status, dominance, prestige, and finance, which might be well demonstrated in an entrepreneurial career. Therefore, previous articles indicated that a high level of psychopathy within individuals positively correlates with start-up intention (Cai et al., 2021; Kramer et al., 2011).

Easily dominated or vulnerable individuals are the target group for people high in psychopathy to take advantage of while giving out insignificant expenditure (Wilson et al., 2008). In addition, power, prestige, and control are beautiful to psychopaths who always want to achieve short-term economic benefits and ignore adverse effects on the environment. Being conscienceless, they do not have a sense of social responsibility as either individual or corporate (Boddy, 2015). To sum up, psychopaths will neglect the interests of related parties and society or the environment to seek their interests. In general, the start-up motives of psychopaths are expected to have a positive orientation toward value-extracting for themselves and a negative association with creating overall value for others as stated in hypotheses H3 (a, b, c) following.

#### H3

*Individuals’ levels of psychopathy have a positive impact on their start-up intention (H3a), selfish start-up motives (H3b), and a negative impact on pro-social start-up motives (H3c).*

## Methodology

### Sample and data

The research investigates the relationship between three selfish personalities of the Dark Triad and start-up intentions of university students in Vietnam. An online survey questionnaire was sent to students via Google Form. The survey was conducted within 2 months from 1st January to 5th March 2021, with 454 responses. After filtering the data, 54 responses were eliminated, because attendants chose the same answers for all the statements, leaving 400 usable cases. The respondents were from 18 universities around the country. Confirmatory factor analysis (CFA) and structured equation modeling (SEM) were used to analyze the final data.

### Measurement

The research utilized the five-point Likert scale for measurement. The range was from 1 (strongly disagree) to 5 (strongly agree). The questionnaire includes 28 measurement variables about the Dark Triad, which were referenced from Hmieleski and Lerner (2016), Jonanson and Webster (2010), and Liñán and Chen (2009).

The scales of measurement were inherently built and developed based on previous research results (Hmieleski & Lerner, 2016; Jonason & Webster, 2010; Liñán & Chen, 2009). The authors then made further adjustments to the questionnaire by translating it into Vietnamese. Interviews with 3 experts who have experience in teaching and studying fields related to university students and education were conducted to test the appropriateness of the Vietnamese version. The researchers also sent the questionnaire to 20 participants and got feedback to make the survey suitable for the target participants.

The three personalities of narcissism, Machiavellianism, and psychopathy were measured by the 12-sentence questionnaire of Jonanson and Webster (2010). Questions to measure narcissism include “I tend to want others to admire me;” “… pay attention to me;” “… expect special favors from others” and “… seek prestige or status”. Machiavellianism was measured by asking participants if they deceive or lie, manipulate, use flattery or exploit others for their own goals. Finally, the psychopathy aspect mentioned attributes of “lacking remorse”, “unconcerned with morality”, “callous” and “cynical”. Hmieleski and Lerner (2016) confirmed Jonanson and Webster’s results, with Cronbach’s coefficient alpha scores of the three constructs ranging from 0.71 to 0.82, demonstrating the high reliability.

#### Start-up intention

The intentions were assessed by the 6-sentence questionnaire of Liñán and Chen (2009), which asks if the responders “are ready to be an entrepreneur,” “have that goal”, “make efforts to start a firm”, “have the determination or serious thought of it”. The scale has a high reported Cronbach alpha score of 0.943.

#### Start-up motives

The research tested entrepreneurial motives in two aspects: selfish and pro-social motives. The instrument was the 10-sentence questionnaire developed by Hmieleski and Lerner (2016). Each motive was measured by five items. Selfish motivation is reflected in “destructive to society,” “at the cost of employees’ well-being,” “scarifying quality,” “at all costs,” and “outsources to reduce costs,” with reported Cronbach alpha of 0.75 to 0.76. The pro-social motive exhibits in “value for society,” “enrich the lives of people,” “employees value their work,” “adding value to the community”, and “employees valuing the corporate mission as their own” with Cronbach alpha of 0.79 to 0.84.

#### Demographic variables

Many different control demographic variables reportedly influencing start-up intention and motives were used in the research. These are age, gender, and entrepreneurship course (Hmieleski & Lerner, 2016), specialization area of study (Liñán & Chen, 2009) and family background (Altinay et al., 2012).

## Results and discussion

### Descriptive statistics

The sample consists of more females (*n* = 230) than males (*n* = 170). The average age is 20.265 years with a standard deviation of 1.961. People aged 20 to 21 make up most of respondents, accounting for 33.25% and 23.75%, respectively. Only one respondent is at the age of 27, and another is over the age of 45. The age structure is realistic, because Vietnamese normally pursue university education right after finishing high school at age of 18. Overall, a large proportion (42.25%) of respondents are students in business and management; other areas are humanities (11%); tourism and hospitability (10%); science and technology (30.75%), and others (6%). The majority of respondents (82.50%) have not taken part in entrepreneurship courses. There is a small number of students that have families who own businesses, accounting for 18.00%. In business and management, humanities, and hospitality higher education, there is a higher proportion of females than males so the gender structure of the sample is not far from the actual structure of university students in Vietnam (Nguyen, 2019).

### Cronbach’s alpha coefficients

Cronbach's coefficient alpha scores for narcissism and Machiavellianism are 0.813 and 0.851, respectively, higher than scores reported by Hmieleski and Lerner (2016) and Jonason and Webster (2010). For the psychopathy construct, reliability analysis points out the divergence of one item, “I tend to be cynical,” with under-threshold Corrected item-total correlation = 0.234 (< 0.3). After eliminating this item, Cronbach's coefficient increases from 0.735 to 0.801, similar to the result by Hmieleski and Lerner (2016) and Jonason and Webster (2010). This may be due to cultural differences and unfamiliarity of term “cynical” that make the question not well understood by most respondents. To be more specific, Asian cultures, including Vietnam, are predominantly collectivistic and lack ground for a separate, autonomous self (Kawamura, 2012). Therefore, the final construct of psychopathy includes three first items among the original four. All scores are presented in Table 2.

**Table 2:** Cronbach’s coefficient of the final three selfish personalities

Items	Scale mean if item deleted	Scale variance if item deleted	Corrected item-total correlation	Cronbach's alpha if item deleted
Narcissism (NAR) alpha = 0.813				
NAR1	11.38	4.861	0.708	0.727
NAR2	11.41	5.105	0.671	0.746
NAR3	11.54	4.953	0.631	0.766
NAR4	11.16	5.670	0.522	0.813
Psychopathy (PSY) alpha = 0.801				
PSY1	5.59	7.072	0.526	0.694
PSY2	5.85	5.293	0.747	0.624
PSY3	5.81	4.413	0.714	0.669
Machiavellianism (MAC) alpha = 0.851				
MAC1	7.03	9.368	0.620	0.841
MAC2	7.55	8.676	0.738	0.791
MAC3	7.39	9.305	0.670	0.820
MAC4	7.80	8.937	0.741	0.791

Source: SPSS analysis results

### Start-up intention and motivation

Cronbach's coefficient alpha of start-up intention construct is 0.846, lower than 0.943 in the original scale (Liñán & Chen, 2009), but it still presents very high consistency. The pro-social start-up motive construct achieves Cronbach's coefficient alpha of 0.881, a little higher than that of Hmieleski and Lerner (2016). However, for the selfish motive factor, a divergence comes from the item (“Outsource work to reduce costs as much as possible”) with a low Corrected item-total correlation of 0.253 (< 0.3). After eliminating this item based on Hair et al. (2014) guidance, Cronbach's coefficient alpha increases from 0.754 to 0.798, similar to Hmieleski and Lerner’s (2016). Outsourcing seems to be a new notion, especially for people outside business classes, so the respondents possibly misunderstood the question. The final selfish motive construct includes four among the five original items. All scores are exhibited in Table 3.

**Table 3:** Cronbach’s coefficient of final start-up intention and motivation constructs

Items	Scale mean if item deleted	Scale variance if item deleted	Corrected item-total correlation	Cronbach's alpha if item deleted
Start-up intention (INT) alpha = 0.846				
INT1	15.47	19.395	0.420	0.855
INT2	14.86	17.178	0.596	0.826
INT3	14.75	16.089	0.703	0.804
INT4	14.86	16.003	0.772	0.791
INT5	15.01	16.302	0.764	0.793
INT6	14.72	18.131	0.513	0.841
Selfish start-up motives (UNP) apha = 0.798				
UNP1	6.42	5.481	0.528	0.727
UNP2	6.91	5.322	0.724	0.746
UNP3	7.10	5.580	0.674	0.766
UNP4	6.87	5.779	0.538	0.785
Pro-social start-up motives (PRO) alpha = 0.881				
PRO1	17.00	6.411	0.672	0.865
PRO2	16.98	6.102	0.729	0.852
PRO3	16.86	5.974	0.735	0.850
PRO4	16.94	6.033	0.705	0.858
PRO5	16.84	5.923	0.734	0.851

Source: SPSS analysis results

### Confirmatory factor analysis (CFA)

**Fig. 1:**
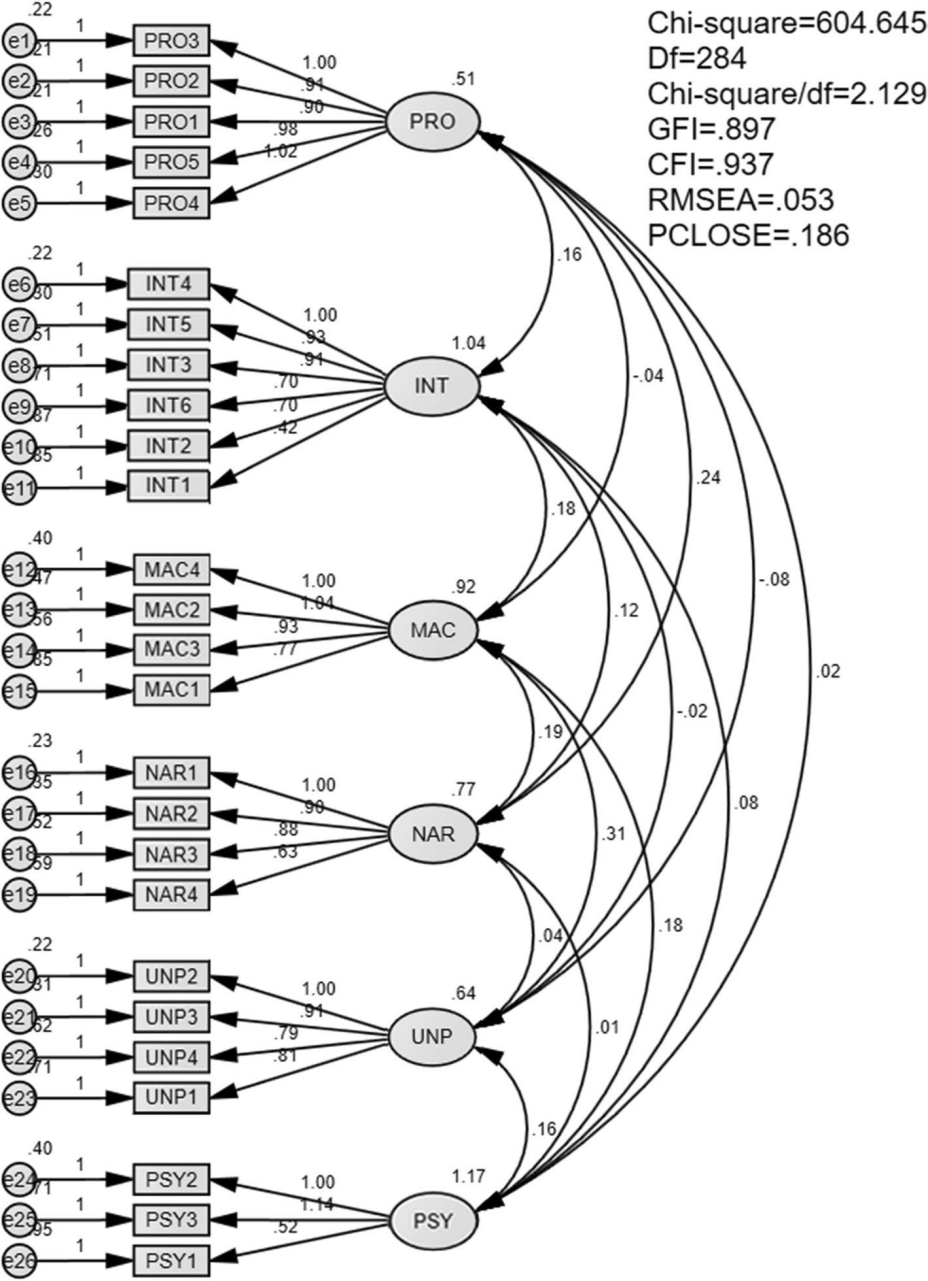
Model of confirmatory factor analysis

The measurement model fit was assessed by CFA procedure using SPSS AMOS, with the final constructs in Tables 2 and 3. The CFA analysis ascertains the suitability of the measurement model with the collected data, in particular: *χ*2 = 604.645, *χ*2/df = 2.129 (good, ≤ 3), RMSEA = 0.053 (good, < 0.06), GFI = 0.897 (acceptable, > 0.8 and approximate 0.9), CFI = 0.937 (good, > 0.9) (see Fig. 1).

Test of discriminant and convergence was conducted based on benchmarks suggested by Hair et al. (2010). As Tables 4 and 5 show, all reliability conditions (Standardized Loading Estimates greater 0.5; Composite Reliability (CR) greater than 0.7); convergent condition (Average Variance Extracted (AVE) greater than 0.5); and discriminant conditions (Maximum Shared Variance (MSV) < Average Variance Extracted (AVE) and Square Root of AVE (SQRTAVE) > Inter-Construct Correlations) are well satisfied. Therefore, the constructs are eligible for further SEM analysis to test the hypotheses.

**Table 4:** Test of discriminant and convergence

	CR	AVE	MSV	MaxR(H)	PRO	INT	MAC	NAR	UNP	PSY
PRO	0.908	0.665	0.155	0.909	**0.815**					
INT	0.864	0.527	0.050	0.916	0.223^***^	**0.726**				
MAC	0.850	0.588	0.159	0.866	−0.052	0.179^**^	**0.767**			
NAR	0.840	0.573	0.155	0.873	0.394^***^	0.140^*^	0.230^***^	**0.757**		
UNP	0.818	0.534	0.159	0.855	−0.136^*^	−0.027	0.399^***^	0.051	**0.731**	
PSY	0.784	0.559	0.034	0.843	0.024	0.072	0.172^**^	0.006	0.184^*^	**0.748**

Bold values present the correlation value (Inter-Construct Correlations) between variables of the correlation matrix

Significance of correlations:

^*^p < 0.050

^**^p < 0.010

^***^p < 0.001

**Table 5:** Standardized loading estimates of constructs

			Estimates (standardized loading)
PRO3	<----	PRO	0.837
PRO2	<---	PRO	0.816
PRO1	<---	PRO	0.817
PRO5	<---	PRO	0.809
PRO4	<---	PRO	0.798
INT4	<---	INT	0.907
INT5	<---	INT	0.866
INT3	<---	INT	0.792
INT6	<---	INT	0.643
INT2	<---	INT	0.605
INT1	<---	INT	0.426
MAC4	<---	MAR	0.835
MAC2	<---	MAR	0.822
MAC3	<---	MAR	0.765
MAC1	<---	MAR	0.628
NAR1	<---	NAR	0.879
NAR2	<---	NAR	0.802
NAR3	<---	NAR	0.731
NAR4	<---	NAR	0.584
UNP2	<---	UNP	0.864
UNP3	<---	UNP	0.793
UNP4	<---	UNP	0.625
UNP1	<---	UNP	0.608
PSY2	<---	PSY	0.863
PSY3	<---	PSY	0.825
PSY1	<---	PSY	0.502

### Structural equation modeling analysis

**Fig. 2:**
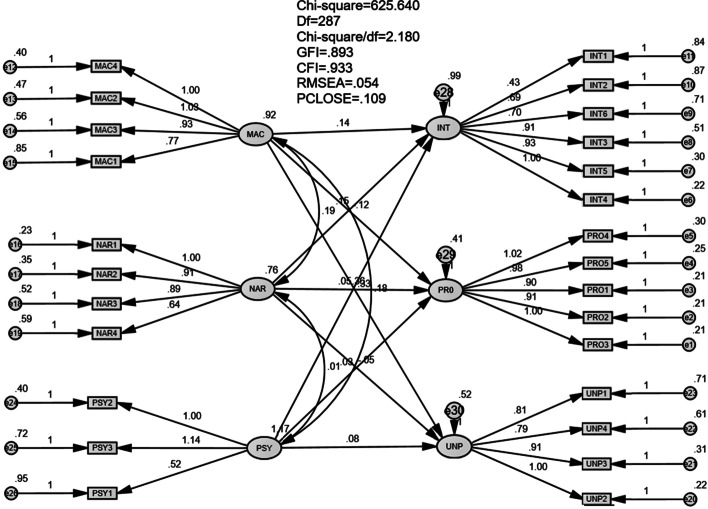
SEM analysis model

The SEM analysis ascertains the suitability of the measurement model with the data, in particular: *χ*2 = 625.640, *χ*2/df = 2.180 (good, ≤ 3), RMSEA = 0.054 (good, < 0.06), GFI = 0.893 (acceptable, > 0.8 and approximate 0.9), CFI = 0.933 (good, > 0.9) (see Fig. 2). Summary of the hypotheses testing results are in Table 6.

**Table 6:** SEM hypotheses testing results

	INT	PRO	UNP
NAR	0.124**	0.436***	−0.050
MAC	0.131**	−0.164**	0.393***
PSY	0.049	0.050	0.115**

Significance levels:

^*^p < 0.050

^**^p < 0.010

^***^p < 0.001

Table 6 shows that narcissism presents no effect on selfish start-up motives (*p* > 0.05); psychopathy presents no effect on both start-up intention and pro-social motives (*p* > 0.05). In other words, hypotheses H1b, H3a, H3c are rejected.

On the other hand, narcissism has a positive association with start-up intentions (*B* = 0.131, *p* < 0.05) and level of pro-social entrepreneurial motives (*B* = 0.436, *p* < 0.01) (that contradicts H1c of negative association). Therefore, hypothesis H1a is supported. Even hypothesis H1c is rejected, it invites further discussion to explain.

As illustrated in Table 6, Machiavellianism presents a positive relationship with start-up intentions (*B* = 0.124, *p* < 0.05) and level of selfish entrepreneurial motives (*B* = 0.393, *p* < 0.01). Besides, a negative relationship is found between Machiavellianism and pro-social start-up motives (*B* = −0.165, *p* < 0.05). These results support hypotheses H2a, H2b and H2c.

As shown in Table 6, psychopathy has only a positive relationship with selfish start-up motives (*B* = 0.115, *p* < 0.05). On the other hand, the construct does not show any link with the other factors. Therefore, hypothesis H3b is supported, while H3a and H3c are rejected.

Analysis of differences among groups demonstrates the influence of entrepreneurship course on start-up intention (students already taking the course having higher intention). Gender and age affect selfish start-up motives (females having lower selfish motives than males, students aged 21 having strongest selfish motives). In addition, family background has an impact on pro-social motives (students with family business backgrounds having lower pro-social motives). All these impacts are significant with *p* < 0.05.

## Discussion

The research demonstrates the diverse impact of the three selfish personalities of the Dark Triad on entrepreneurship. A higher level of narcissism and Machiavellianism leads to stronger start-up intention, while psychopathy shows no such impact. In addition, Machiavellianism has a positive association with selfish entrepreneurial motive and a negative relation with pro-social start-up motive. Furthermore, narcissism is positively related to pro-social entrepreneurship, while psychopathy has a positive relationship with selfish venturing.

According to the life history theory, selfish people with strong Dark Triad personalities that induce them to follow a “fast life” approach will have a greater ambition to become entrepreneurs. However, the effect of the triad is not consistent; only two of them, narcissism and Machiavellianism, present such impact, of which Machiavellianism has a more substantial influence. This result is slightly different from findings by Hmieleski and Lerner (2016), Mathieu and St-Jean (2013), in which only narcissism affects entrepreneurial intention. The reason may come from cultural differences. This research is based on data from an Asian country, while the context of Mathieu and St-Jean, Hmieleski and Lerner’s study is the United States with Western values and a strict legal system. It may be harder to follow a “fast life” in established organizations, such as public organizations and corporations, because it usually requires a long time to get to high positions via a bureaucratic promotion process. In addition, the less developed legal system in Vietnam is offering gaps for making fast money, even in big state-owned banks (Lien & Holloway, 2014). It makes start-up attractive to people high in narcissism and Machiavellianism, helping them become CEO in a short time, instead of years, and making dirty money with little concerns of being punished.

On the other hand, the results show that psychopathy does not have a relationship with entrepreneurial intention. This finding contrasts with Cai et al. (2021) and Kramer et al. (2011), but agrees with Hmieleski and Lerner (2016). Lacking sympathy to other people (Kramer et al., 2011), psychopaths may find it difficult to hide their evil nature in transactions. In start-up context of Vietnam, people tend to work in a team of friends and family members. With long-lasting and close connections, friends can know each other thoroughly, and the opportunities for someone to take advantage of the other are limited. The chance is greater when psychopaths work with strangers. As a result, they may find it more favorable to work as employees for corporations in which they shall not need to worry about people recognizing their intention to exploit co-workers for their purpose.

According to a study, the general callous nature of people high in psychopathy has a negative relation with social-oriented development (Akhtar et al., 2013). They are willing to go against social standards and benchmarks (Mathieu & St-Jean, 2013). When it comes to entrepreneurial activities, psychopaths also have the intention to disrespect corporate social responsibility (CSR); they are likely to be an obstacle to the continuous effort required for creating social value and innovation of a venture. The other end of the pro-social motive is the selfish motive (Van Kleef & De Dreu, 2002). Agreeing with this logic, this research found no significant association between psychopathy and pro-social start-up motives, but a positive relation with selfish motives. The psychopaths are aware that they are living in a collectivist culture of Vietnam; to be safe, they may take advantage of other people when opportunities are available, but they do not go against activities for the common good, so that people still accept their appearance.

Narcissism shares the feature of a grandiose sense of self-worth with psychopathy (Hare, 1991), which leads to numerous impulsive and irresponsible actions without considering the possible negative consequences. In contrast with this prediction but agreeing with Hmieleski and Lerner (2016) and O’Reilly and Pfeffer (2021), the finding shows that narcissistic people are more likely to have pro-social start-up motives. This research’s data were collected during the time of the COVID-19 pandemic in Vietnam. During such a difficult time, the normal respect of rich people gives way for the admiration to people who can help the community by distributing essential goods to the poor, devising robots for distant distribution, inventing reusable face masks in the context of shortage, etc. The motivation to be recognized quickly induces the narcissism to divert their attention to pro-social start-up. O’Reilly and Pfeffer (2021) investigated that people who are high in narcissism are more likely to perform behaviors aligned with organizational strategies and norms to achieve goals within their profession. Furthermore, narcissists are engaged with value creation rather than value appropriation, because pro-social start-up motives are predicted to benefit their relationships and lives highly.

Not only promoting start-up intention, but Machiavellianism also encourages people to engage in selfish motives and avoid pro-social motives. While Hmieleski and Lerner (2016) found only the first relation, this finding lends more support to the long-existing notion that Machiavellians are economic opportunistic (Sakalaki et al., 2007). The pro-social start-ups may be in the form of social enterprises whose profitability is normally lower than ordinary businesses or need real value contributions via innovation that requires a long time to develop. The Machiavellians are inclined to quick gain, so they are not interested in a vision of long-term gain. As a result, they avoid it. On the other hand, scamming and cheating other people seen in businesses with selfish motives can help the owners get quick outcomes, which are attractive to the Machiavellians.

To sum up, among the three selfish traits of the Dark Triad, Machiavellianism demonstrates the most consistent impact on all start-up intentions and two types of motives as predicted. The next significant trait is narcissism which has a predicted influence on entrepreneurial intentions; however, it has an unexpected positive impact on pro-social start-up motives. Finally, the last dark personality of psychopathy only anticipates selfish motives and shows no significant effects on entrepreneurial intentions and pro-social motives. Implications of the findings are presented below.

## Implications, limitations, and future directions

### Implications

Affected by the culture of community belonging and collectivist ideals, Vietnamese students are likely to partake in peer comparisons to guarantee that they are adhering to norms and standards. Selfish people with the Dark Triad are sometimes perceived as negative standout or divergence, which can cause “face-losing” (Kawamura, 2012); however, they possess a solid tendency to entrepreneurship. Therefore, for educators, boycotting students with either narcissism, psychopathy, or Machiavellianism should be prohibited. In fact, such people are perceived as the next entrepreneur-generation and valuable intangible resources of any country or region. Recognizing the trend of global self-employment, universities are vital for establishing social foundations and entrepreneurial ecosystems for future economic growth (Blaese et al., 2021). Therefore, entrepreneurship programs should be considered basic courses for business undergraduates, thanks to which students are trained to have moral and appropriate attitudes, critical skills, and information (Chen et al., 1998). Furthermore, self-regulation and social responsibility should be developed to reduce the harmful effects of dark characteristics on humanity and business (Cai et al., 2021; Hmieleski & Lerner, 2016).

Vietnamese education is said to be ‘spoon-feeding,’ making students follow surface learning strategies (Ramsden, 1992) to deal with tests and achieve short-term success instead of fully absorbing knowledge. The fierceness of high-school entrance exams, and students’ lacking survival skills, while spending most of the time in classes is an example. That kind of education is favorable for characteristics of Machiavellianism and narcissism. Therefore, innovative teaching is needed to encourage new learning methods (e.g., self-learning), appropriate learning environments, especially in business schools. Nguyen (2018) urged governments and higher education institutions should offer entrepreneurship training services to everyone who wants to start a company, not just people with a high school diploma. Entrepreneurship should be promoted in every educational setting, not just in traditional institutions, such as universities or colleges.

In a start-up ecosystem, investors may follow either profit or impact direction. For the impact funds, whose principal purpose is to promote pro-social start-ups, managers should have methods to screen founders’ personalities. Narcissistic founders are eligible, because they are interested in pro-social motives. However, care should be taken when dealing with psychopaths or the Machiavellians, because these people are prone to selfish businesses and avoid engaging in pro-social causes.

## Limitations and future research

The research has some limitations, which can be recognized as research gaps for future studies. The first limitation is the data collection method and sample size. The authors have used convenience sampling for reaching out to respondents, which has a risk of reduced credibility (Leiner, 2016). Although the population is Vietnam undergraduates, most of the respondents came from easily reached universities. Further research should study all Vietnam universities and increase the sample size by attracting more respondents with creative data collection methods, which achieve better credibility and generalization. In addition, participants should be expanded to working people to increase generalization of findings.

Another limitation is vague questions. The survey questionnaire is translated into Vietnamese for better respondents’ understanding and convenience. However, two of the questions are not well understood by respondents. For instance, the question “I tend to be cynical” might be unfamiliar with the collectivist Vietnamese individuals; hence it misled the answer of respondents. Moreover, among questions to measure entrepreneurial motives, the statement “Outsource work to reduce costs as much as possible” contains an unfamiliar term of “outsource” to respondents, so that the results might not truly reflect the respondents’ motives. Therefore, further research should ensure all the questions are better grasped and understood by respondents, considering cultural distinction and terminology.

The impact of the three selfish personalities on entrepreneurship is mixed in terms of significance and directions of relationships. While not interested in starting a business and pro-social start-up motives, psychopaths are associated with selfish entrepreneurial motives. On the other hand, narcissists are involved with pro-social motives but show no significant linkage with selfish motives, contradicting theories. This is a big gap for future studies to investigate.

The final limitation is the cross-sectional design that limits the investigation to start-up intention only. Therefore, further research is advised to survey the influence of dark characteristics on entrepreneurial behaviors instead of intention.

## Conclusions

Machiavellianism is the best predictor in the research model, because it has a significant predicted impact on startup intention, pro-social motives and selfish motives. While narcissism shows a significant relationship with both start-up intentions and pro-social motives, psychopathy only affects selfish start-up motives. These findings share consistency as well as some contradictions with other studies conducted in Western countries. The discrepancies are explained by typical social-cultural conditions in Vietnam—an Asian country, making the differences contributions to entrepreneurship literature. Finally, the research promotes a multidimensional perspective of entrepreneurs, who have both good side and dark side, to encourage more objective and appropriate decision-making in business education and venture capital management, by better recognizing who entrepreneurs are.

## Data Availability

The data set used during the current study are available from the corresponding author on request.
